# Translational Animal Models in Colitis: From Rodents to Pig and Minipig Platforms

**DOI:** 10.3390/ijms27125414

**Published:** 2026-06-16

**Authors:** Woon Kyu Lee

**Affiliations:** Department of Biomedical Sciences, School of Medicine, Inha University, Incheon 22212, Republic of Korea; wklee@inha.ac.kr; Tel.: +82-32-860-9882; Fax: +82-32-885-8302

**Keywords:** inflammatory bowel disease, colitis model, porcine model, minipig, translational medicine, ulcerative colitis, Crohn’s disease

## Abstract

Inflammatory bowel disease (IBD), including ulcerative colitis (UC) and Crohn’s disease (CD), is a chronic relapsing inflammatory disorder characterized by epithelial barrier dysfunction, immune dysregulation, microbiota imbalance, and progressive tissue remodeling. Because the pathogenesis of IBD involves complex interactions among genetic, immunological, microbial, and environmental factors, experimental animal models have become indispensable tools for investigating disease mechanisms and evaluating therapeutic strategies. Various experimental colitis models have been developed to reproduce distinct pathological features of human IBD, including chemically induced models, genetically engineered systems, adoptive immune-transfer models, and infectious or microbiota-associated models. Rodent models remain the most widely used experimental platforms because of their accessibility, reproducibility, and well-established genetic manipulation technologies. These systems have significantly contributed to understanding inflammatory signaling pathways, epithelial barrier injury, immune cell dysregulation, and gut microbial crosstalk. However, important species-specific differences in intestinal anatomy, immune responses, microbiota composition, and pharmacokinetics limit direct translation of rodent findings into clinical applications. To overcome these limitations, increasing attention has been directed toward large-animal models, particularly pig and minipig systems, which more closely resemble human gastrointestinal anatomy, digestive physiology, immune regulation, and microbiome-related characteristics. Porcine models additionally support clinically relevant procedures, including repeated colonoscopy, serial biopsy sampling, pharmacokinetic evaluation, and longitudinal therapeutic monitoring. Recent advances in genome-editing technologies and multi-omics approaches have further enhanced the translational utility of porcine IBD models. This review summarizes major experimental colitis animal models, discusses their pathological and translational characteristics, and highlights the growing importance of pig and minipig systems as human-applicable platforms for preclinical therapeutic evaluation and translational IBD research.

## 1. Introduction

Inflammatory bowel disease (IBD), including ulcerative colitis (UC) and Crohn’s disease (CD), is a chronic relapsing inflammatory disorder of the gastrointestinal tract characterized by epithelial barrier dysfunction, immune dysregulation, and alterations in the gut microbiota [1,2,3,4]. Although animal models have contributed substantially to understanding disease mechanisms and therapeutic development, no single model fully reproduces the complexity of human IBD [5,6,7]. Because each animal model reproduces only selected aspects of human disease, careful model selection is essential for accurate experimental interpretation and improved clinical relevance in IBD research [8,9,10].

Rodent models remain the most widely used systems because of their accessibility, reproducibility, and genetic manipulability. However, important anatomical and physiological differences from humans limit their clinical applicability, particularly for long-term therapeutic evaluation and clinically applicable procedures [10]. To address these limitations, increasing attention has been directed toward large-animal models, especially pigs and minipigs, which more closely resemble human gastrointestinal anatomy, immune responses, and metabolic characteristics [11,12,13]. These models enable repeated endoscopy, longitudinal biopsy, and longitudinal therapeutic assessment [14]. Unlike previous reviews that primarily focus on rodent colitis models, this review emphasizes the translational limitations of conventional small-animal systems and highlights the emerging role of pig and minipig models as clinically meaningful platforms. Particular attention is given to endoscopic evaluation, longitudinal biopsy sampling, pharmacokinetic assessment, genome-edited porcine models, and their emerging applications in translational IBD research and drug development.

## 2. Pathophysiological Features That Should Be Reflected in Colitis Models

An ideal experimental colitis model should reproduce the major pathological and immunological characteristics of human IBD, including epithelial barrier dysfunction, mucosal ulceration, inflammatory cell infiltration, cytokine dysregulation, microbiota imbalance, and chronic tissue remodeling [15,16,17]. Although UC and CD share several inflammatory features, their pathological characteristics differ substantially. UC is generally characterized by continuous mucosal inflammation confined to the colon, whereas CD involves transmural inflammation, skip lesions, fibrosis, strictures, and fistula formation [16,17].

Disruption of the intestinal epithelial barrier is considered one of the earliest and most critical events in IBD pathogenesis [18]. The intestinal barrier is composed of a mucus layer, epithelial cells, antimicrobial peptides, immune components, and intercellular tight junction complexes that collectively regulate intestinal permeability and maintain mucosal homeostasis. Tight junction proteins, including zonula occludens-1 (ZO-1), occludin, and members of the claudin family, play pivotal roles in preserving epithelial integrity and preventing excessive translocation of luminal antigens and microorganisms across the intestinal mucosa. Loss of barrier function has been consistently associated with increased intestinal permeability, enhanced microbial translocation, aberrant immune activation, and perpetuation of chronic intestinal inflammation [15,16,18].

Both innate and adaptive immune responses play central roles in the initiation and chronicity of intestinal inflammation [5,19]. Activation of macrophages, dendritic cells, neutrophils, and innate lymphoid cells promotes excessive production of inflammatory mediators, including TNF-α, IL-1β, IL-6, IL-17, and interferon-γ. Dysregulation of adaptive immune pathways, particularly the imbalance among Th1, Th2, Th17, and regulatory T-cell populations, further amplifies chronic inflammatory responses [20]. CD is generally associated with Th1- and Th17-dominant immune activation, whereas UC more commonly exhibits atypical Th2-associated inflammation. Persistent cytokine activation contributes not only to mucosal injury but also to fibrosis, tissue remodeling, and progressive loss of intestinal homeostasis [5,20].

Alterations in the intestinal microbiota are also recognized as major contributors to IBD pathogenesis [21]. Dysbiosis is characterized by reduced microbial diversity, depletion of beneficial commensal bacteria, and expansion of pro-inflammatory microbial populations. These microbial alterations influence epithelial integrity, immune signaling, and metabolic homeostasis through interactions involving microbial metabolites, pattern-recognition receptors, and inflammatory signaling pathways [22,23]. In addition, oxidative stress, mitochondrial dysfunction, and endoplasmic reticulum stress further contribute to epithelial injury and chronic inflammation [16,18].

Because human IBD is multifactorial and highly heterogeneous, no single experimental model can fully reproduce all pathological and immunological aspects of the disease [8,9,10]. Consequently, different experimental systems reproduce distinct components of disease progression and inflammatory responses. DSS-induced colitis primarily reflects epithelial injury and innate immune activation, whereas TNBS and adoptive T-cell transfer models better reproduce T-cell-mediated persistent inflammatory responses [8,9,24]. Genetically engineered models, including IL-10 knockout mice, are particularly valuable for investigating aberrant immune activation, microbiota-associated inflammation, and chronic intestinal pathology [25]. Therefore, appropriate model selection should be guided by the specific mechanistic and translational objectives of each study.

## 3. Classification of Colitis Animal Models

### 3.1. Chemically Induced Models

Chemically induced colitis models are among the most widely utilized experimental systems in inflammatory bowel disease (IBD) research because of their technical simplicity, reproducibility, and rapid disease induction [26,27,28]. Among these, dextran sulfate sodium (DSS)-induced colitis is the most extensively used model and primarily reflects epithelial barrier disruption and innate immune activation associated with ulcerative colitis-like pathology [29]. DSS damages epithelial cells and compromises mucosal barrier integrity, resulting in increased intestinal permeability, microbial translocation, and subsequent activation of innate and adaptive inflammatory pathways. Typical pathological features include weight loss, diarrhea, rectal bleeding, crypt damage, epithelial ulceration, and inflammatory cell infiltration [15,18]. Repeated DSS administration can further induce chronic inflammation, fibrosis, and tissue remodeling [26]. However, disease severity is influenced by several experimental variables, including DSS molecular weight, concentration, treatment duration, animal strain, microbiota composition, and environmental conditions [29].

Other chemically induced models reproduce distinct aspects of IBD pathology. TNBS- and DNBS-induced colitis involve hapten-mediated immune activation and generate Th1/Th17-dominant transmural inflammation resembling several features of Crohn’s disease [26,30]. Chronic exposure may additionally promote fibrosis and intestinal remodeling, making these models useful for investigating immune-mediated inflammation and anti-fibrotic therapies. Oxazolone-induced colitis is characterized by Th2-associated mucosal inflammation and superficial epithelial injury that resembles selected immunological aspects of ulcerative colitis, whereas acetic acid models rapidly induce epithelial ulceration and acute mucosal injury but have limited applicability for chronic disease studies [30,31]. Although chemically induced models remain highly valuable for mechanistic investigations and therapeutic screening, they do not fully reproduce the multifactorial and heterogeneous nature of human IBD [26,28]. The major characteristics, advantages, and limitations of commonly used chemically induced colitis models are summarized in Table 1.

### 3.2. Genetically Engineered Models

Genetically engineered models provide important insights into the molecular and immunological mechanisms underlying chronic intestinal inflammation [26,28]. IL-10 knockout mice are among the best-characterized models and develop spontaneous microbiota-dependent enterocolitis caused by impaired immune regulation [25]. Other models, including IL-2, TCR, and NOD2-associated systems, are useful for investigating immune dysfunction and host–microbiota interactions relevant to IBD [32,33]. These systems are particularly useful for mechanistic investigation and target validation. However, disease phenotype and severity are strongly influenced by genetic background, environmental conditions, and microbiota composition, which may limit reproducibility and translational utility [20,26,28].

### 3.3. Adoptive Transfer Models

Adoptive T-cell transfer models are widely used to investigate T-cell-mediated chronic intestinal inflammation [34,35]. Transfer of naïve CD4+ CD45RB^high^ T cells into immunodeficient mice induces progressive colitis characterized by chronic mucosal inflammation and cytokine dysregulation. These models are particularly useful for studying effector and regulatory T-cell balance and evaluating immune-targeted therapies [36]. Compared with chemically induced models, adoptive transfer systems better reflect chronic immune dysregulation. However, the artificial nature of immune reconstitution and the use of immunodeficient recipients limit direct clinical relevance [8,28].

### 3.4. Infectious and Microbiota-Associated Models

**Figure 1:**
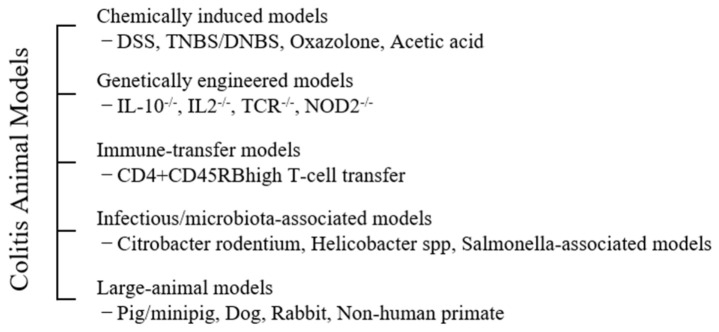
Classification and translational characteristics of experimental colitis animal models.

Infectious- and microbiota-associated models are useful for studying host–microbe interactions in intestinal inflammation [21,37]. Representative systems include Citrobacter rodentium, Helicobacter species, and Salmonella infection models, which induce inflammation through microbial-driven immune activation and epithelial disruption [37,38]. These models are valuable for investigating dysbiosis, barrier function, and host–microbiota crosstalk [21,37]. However, because inflammation is induced by defined pathogens, they do not fully reproduce the multifactorial and idiopathic nature of human IBD [8,28]. A comparative overview of experimental colitis animal models and their representative pathological characteristics is presented in Figure 1.

Experimental colitis models include chemically induced, genetically engineered, adoptive immune-transfer, infectious/microbiota-associated, and large-animal systems. Each model reproduces specific aspects of IBD, including epithelial injury, immune dysregulation, fibrosis, and host–microbiota interactions. Rodent models remain indispensable for mechanistic studies and therapeutic screening, whereas pig and minipig models provide enhanced translational relevance through clinically applicable procedures such as endoscopy, serial biopsy sampling, pharmacokinetic evaluation, and longitudinal disease monitoring.

## 4. Rodent Models: Strengths and Limitations

Rodent models have played a central role in advancing our understanding of IBD and continue to serve as indispensable tools for mechanistic investigations [10,39]. Together, chemically induced, genetically engineered, adoptive transfer, and microbiota-associated models provide complementary approaches for investigating epithelial barrier dysfunction, immune dysregulation, host–microbiota interactions, and inflammatory signaling pathways involved in intestinal inflammation [8,10]. A major strength of rodent systems lies in their ability to dissect disease mechanisms through precise genetic and immunological manipulation. Studies using transgenic, knockout, and microbiota-modified models have substantially contributed to the identification of pathways associated with cytokine signaling, epithelial barrier integrity, immune regulation, and host–microbial interactions in IBD [5,8,21].

From a practical perspective, rodent systems remain highly advantageous for early-stage therapeutic development because disease induction is relatively rapid and experimental conditions can be tightly controlled. DSS-induced models are widely used for screening anti-inflammatory and epithelial-protective agents, whereas TNBS and adoptive transfer systems are more suitable for evaluating chronic immune-mediated inflammation and immune-modulating therapies [26,29,30,35]. Genetically engineered mouse models additionally provide important platforms for mechanistic target validation and investigation of disease-associated susceptibility genes and molecular pathways implicated in human IBD [8,33].

Despite their indispensable role in mechanistic research, rodent models possess several important limitations that restrict direct clinical translation. Significant interspecies differences exist in gastrointestinal anatomy, immune-cell composition, microbiota structure, metabolic physiology, and pharmacokinetic responses between rodents and humans [11,12,13]. Furthermore, the small body size of rodents limits the application of clinically relevant procedures, including repeated colonoscopy, serial biopsy sampling, advanced imaging, pharmacokinetic/pharmacodynamic (PK/PD) evaluation, and device-based therapeutic interventions [14]. These limitations are particularly important when assessing long-term therapeutic efficacy, mucosal healing, safety profiles, and translational biomarkers [13,14,40].

As the therapeutic landscape of inflammatory bowel disease increasingly expands toward biologics, microbiome-targeted therapies, regenerative medicine, and endoscopic interventions, there is growing demand for preclinical models that more closely reflect human physiology and clinical practice [5,20]. Consequently, large-animal systems, particularly pig and minipig models, have emerged as promising translational platforms because they enable human-scale procedures, longitudinal disease monitoring, and clinically meaningful therapeutic evaluation [13,14]. Rather than replacing rodent models, these systems should be viewed as complementary platforms that bridge the gap between mechanistic discoveries and clinical application.

## 5. Large Animal Models of Colitis

### 5.1. Pig and Minipig Models

Pig and minipig models have emerged as highly valuable translational platforms in inflammatory bowel disease (IBD) research because of their close anatomical, physiological, immunological, and metabolic similarity to humans [11,13,14]. Compared with conventional rodent systems, pigs exhibit gastrointestinal characteristics that more closely resemble the human intestine, including comparable intestinal length, mucosal architecture, digestive physiology, dietary behavior, and microbiota composition [41]. Porcine intestinal tissues also possess well-developed Peyer’s patches, mucus-producing epithelial structures, and immune-related signaling pathways with relatively high homology to humans, supporting their utility as clinically relevant immunological models [11,12,42,43].

One of the major advantages of pig and minipig systems is their suitability for human-scale procedures and longitudinal disease monitoring [13,14]. Their larger body size enables the use of standard clinical instruments and therapeutic devices that are difficult to implement in rodents. Repeated colonoscopic evaluation allows direct assessment of mucosal inflammation, ulceration, edema, vascular alteration, spontaneous bleeding, and mucosal healing during disease progression and therapeutic intervention. In addition, serial biopsy sampling enables longitudinal histopathological, molecular, and microbiome-related analyses within the same animal, thereby reducing experimental variability and improving clinical applicability [42,44].

Among currently available porcine models, DSS-induced colitis is the most widely used chemically induced system for reproducing ulcerative colitis-like pathology [45,46]. Administration of DSS in drinking water induces epithelial barrier disruption, increased intestinal permeability, mucosal ulceration, inflammatory cell infiltration, diarrhea, and rectal bleeding. Endoscopic examination typically reveals mucosal erythema, edema, friability, and ulcerative lesions, whereas histological evaluation demonstrates crypt distortion, epithelial erosion, goblet-cell depletion, and mucosal inflammatory changes [45,46]. Recent studies have further shown that DSS exposure alters gut microbial composition and metabolite profiles, supporting its utility for investigating host–microbiota interactions and microbiome-targeted therapeutic strategies [45]. Importantly, the larger body size of pigs permits repeated endoscopic monitoring and serial tissue collection, providing advantages for longitudinal assessment of disease progression and therapeutic response that are difficult to achieve in rodent models [42,44].

TNBS- and DNBS-induced porcine colitis models have also been developed to reproduce pathological features resembling Crohn’s disease. These models induce transmural inflammation through hapten-mediated immune activation and are associated with inflammatory cell infiltration extending beyond the mucosal layer, tissue remodeling, and fibrosis [45,46,47]. Compared with DSS-induced colitis, TNBS/DNBS models are particularly useful for studying chronic inflammatory responses, fibrotic progression, and evaluation of anti-fibrotic therapies. Furthermore, the porcine gastrointestinal tract allows clinically meaningful assessment using colonoscopy, imaging modalities, and repeated biopsy sampling, thereby facilitating translational evaluation of therapeutic interventions under conditions that more closely resemble clinical practice.

Recent advances in genome-editing technologies have accelerated the development of genetically engineered porcine IBD models. Among these, TNFΔARE pigs represent one of the most promising translational platforms currently available [48]. These animals develop spontaneous chronic intestinal inflammation caused by dysregulated TNF expression and exhibit pathological features that closely resemble human Crohn’s disease, including epithelial barrier dysfunction, transmural inflammation, immune-cell infiltration, and altered microbial composition. Unlike chemically induced systems, TNFΔARE pigs provide opportunities to investigate long-term disease progression, chronic immune dysregulation, and treatment responses in a genetically defined setting. Consequently, they are increasingly regarded as valuable models for translational studies evaluating biologics, microbiome-based interventions, and precision-medicine approaches.

The translational utility of porcine models is expected to continue expanding with advances in microbiota-humanized pigs, gene-editing technologies, single-cell and spatial multi-omics analyses, and AI-assisted digital pathology. These innovations will enable more precise characterization of disease mechanisms, patient heterogeneity, and therapeutic responses, thereby enhancing the predictive value of preclinical studies. Although challenges related to cost, infrastructure requirements, experimental throughput, and protocol standardization remain, ongoing technological and methodological improvements are likely to increase the accessibility and reproducibility of porcine IBD models. Consequently, pig and minipig systems are increasingly recognized as indispensable translational platforms that bridge the gap between rodent studies and human clinical trials, accelerating the development of next-generation therapies and precision medicine approaches for IBD [49,50,51,52,53]. The major characteristics, translational applications, and limitations of currently available porcine colitis models are summarized in Table 2.

### 5.2. Other Large-Animal Models

Other large-animal models, including dogs, rabbits, and non-human primates, have also been explored in intestinal inflammation research and translational therapeutic studies [54,55,56]. However, their broader application is restricted by high cost, ethical concerns, and limited standardization.

Compared with these systems, pigs and minipigs provide a more practical balance between clinical applicability and experimental feasibility. Consequently, they are increasingly regarded as the most suitable large-animal platforms for bridging preclinical and clinical IBD research. Key differences between rodent and pig/minipig colitis models are summarized in Table 3.

## 6. Evaluation Methods in Colitis Models

Evaluation of experimental colitis models requires integrated assessment of clinical, histopathological, molecular, microbiological, and imaging-based parameters, as no single endpoint adequately reflects disease severity or treatment response [57]. Clinical indicators commonly include body weight loss, stool consistency, rectal bleeding, colon shortening, and disease activity index (DAI) scoring [58,59]. Macroscopic evaluation additionally assesses mucosal edema, ulceration, hemorrhage, and tissue thickening associated with inflammatory progression [57].

Histopathological analysis remains one of the most important approaches for assessing inflammatory severity and tissue damage in experimental colitis models [58,59]. Histological scoring systems typically evaluate epithelial injury, inflammatory cell infiltration, crypt destruction, goblet cell depletion, edema, and ulceration. In chronic models, additional assessment of fibrosis, muscular thickening, and tissue remodeling is often required to characterize long-term pathological progression [60]. Colonoscopic and advanced imaging techniques may further provide direct visualization of mucosal ulceration, vascular pattern alteration, spontaneous bleeding, and mucosal healing during disease progression and therapeutic intervention [61,62].

Molecular analyses commonly include quantitative assessment of inflammatory cytokines and chemokines, including TNF-α, IL-6, IL-17, and interferon-γ, together with epithelial barrier markers such as ZO-1 and occludin [5,63]. Oxidative stress markers, apoptosis-related pathways, and signaling molecules associated with immune activation and tissue repair are also frequently evaluated [64]. In addition, microbiome analyses provide important insight into dysbiosis and host–microbe interactions contributing to intestinal inflammation [22].

Large-animal models provide additional advantages for translational evaluation because they support repeated endoscopic assessment, longitudinal biopsy sampling, serial blood collection, and human-applicable imaging procedures [11,14]. These approaches enable continuous monitoring of disease progression, therapeutic response, and mucosal healing within the same animal, thereby improving the clinical applicability of preclinical IBD studies [14].

Recent advances in multi-omics technologies have substantially expanded the analytical scope of experimental colitis research. Transcriptomics, proteomics, metabolomics, microbiome profiling, and spatial transcriptomics provide comprehensive insight into disease-associated molecular networks and host–microbial interactions [52,65,66]. In addition, AI-assisted digital pathology and computational image analysis are emerging as promising approaches for improving objective histopathological evaluation and translational predictability in preclinical IBD research [67].

Importantly, the most informative evaluation endpoints vary according to the pathological characteristics and translational objectives of each experimental model [57]. DSS-induced colitis is particularly suitable for assessing epithelial barrier integrity and mucosal healing, whereas TNBS/DNBS models are more informative for evaluating transmural inflammation and fibrosis [58,68]. Genetically engineered models are valuable for cytokine profiling and immune dysregulation studies [8], while pig and minipig models enable clinically relevant assessment through endoscopy, serial biopsy sampling, imaging, and PK/PD analyses [11,14]. Representative evaluation strategies according to model type are summarized in Table 4.

## 7. Applications in Drug Development

Experimental colitis models are widely used for evaluating anti-inflammatory drugs, biologics, microbiome-targeted therapies, stem cell therapies, and regenerative approaches for inflammatory bowel disease (IBD) [28]. Chemically induced models, particularly DSS-induced colitis, are commonly utilized for rapid screening of anti-inflammatory and epithelial-protective agents because of their reproducibility and relatively simple induction protocols [69]. In contrast, TNBS and adoptive transfer models are more suitable for evaluating immune-modulating therapies because they better reproduce chronic immune-mediated inflammation associated with Crohn’s disease-like pathology [8,68].

Genetically engineered models are particularly valuable for mechanistic analysis and target validation during early-stage drug discovery [8]. These systems have contributed substantially to identifying therapeutic targets associated with cytokine signaling, epithelial barrier dysfunction, and microbiota-associated inflammation [70,71]. Rodent models additionally provide practical advantages for preclinical screening because disease induction is relatively rapid and experimental conditions can be tightly controlled [26,28].

The expanding therapeutic landscape of IBD has extended the use of experimental colitis models beyond conventional anti-inflammatory drug screening [5]. Several biologic agents currently used in clinical practice, including the anti-TNF monoclonal antibody infliximab, the anti-integrin antibody vedolizumab, and the anti-IL-12/23 antibody ustekinumab, were initially evaluated in preclinical models that provided important insights into cytokine regulation, leukocyte trafficking, and mucosal immune responses [72]. Experimental colitis models have also been widely utilized for assessing emerging therapeutic targets involving JAK/STAT signaling, epithelial barrier restoration, and immune-cell modulation [72,73].

In parallel, microbiome-based therapeutic strategies, including probiotics, fecal microbiota transplantation (FMT), and microbial metabolite modulation, have attracted increasing attention as potential approaches for restoring intestinal homeostasis. Rodent models remain valuable for mechanistic studies of host–microbiota interactions, whereas large-animal systems, particularly pig and minipig models, may provide additional translational advantages because of their closer resemblance to human gastrointestinal physiology, microbiota composition, and therapeutic administration protocols. These models are increasingly being utilized to evaluate microbiome-targeted interventions, biologics, regenerative therapies, and advanced drug-delivery systems under clinically meaningful conditions [74,75,76,77].

Stem cell therapies, extracellular vesicle-based therapeutics, and biomaterial-assisted local drug delivery systems are also being investigated to improve mucosal regeneration and long-term disease control [78,79,80]. Because these approaches often require long-term efficacy and safety assessment, large-animal systems may provide additional advantages for evaluating therapeutic dosing strategies, delivery efficiency, pharmacokinetics, and longitudinal mucosal healing [11,14]. Integrating mechanistic rodent systems with clinically feasible large-animal platforms may therefore improve the translational reliability of preclinical IBD research and support more efficient therapeutic development [14].

## 8. Limitations and Future Perspectives

Current experimental colitis models remain limited in their ability to fully reproduce the complexity and heterogeneity of human inflammatory bowel disease (IBD) [28,70]. Chemically induced systems primarily reflect acute epithelial injury and innate immune activation, whereas genetically engineered and adoptive transfer models incompletely reproduce the complex interactions among immune regulation, microbiota imbalance, environmental exposure, and chronic tissue remodeling associated with human disease [8,57]. Consequently, no single experimental platform can fully represent the pathological diversity and clinical progression of IBD [64,71].

Although rodent models remain indispensable for mechanistic analysis and early-stage therapeutic screening, their translational limitations highlight the need for clinically applicable large-animal platforms [8,28]. In particular, standardized pig and minipig models capable of reproducing both ulcerative colitis- and Crohn’s disease-like pathology remain an important unmet need in translational IBD research [81,82,83,84,85].

Future progress in experimental colitis research will likely depend on further improvements in reproducibility, standardization, and translational applicability through the integration of endoscopic assessment, longitudinal biopsy analysis, histopathological scoring, and multi-omics approaches [57,59,65]. These strategies may improve the predictive reliability of preclinical models and enable more comprehensive evaluation of disease progression and treatment response [67].

Emerging technologies, including single-cell RNA sequencing, spatial transcriptomics, microbiome profiling, patient-derived organoid systems, and AI-assisted digital pathology, are expected to complement conventional animal models and provide deeper insight into cellular heterogeneity and tissue-specific inflammatory responses. Such integrative approaches may facilitate biomarker discovery, objective disease scoring, and precision medicine strategies tailored to individual inflammatory and microbiota-associated disease phenotypes [52,65,66,67,86].

Despite ongoing technological advances, several challenges remain unresolved. Variability in induction protocols, microbiota conditions, dietary factors, environmental exposure, and inter-laboratory reproducibility continues to limit broader translational application of large-animal IBD models [57,59]. Therefore, future efforts should focus not only on mechanistic refinement but also on establishing standardized and clinically predictive experimental platforms [65]. Integrating mechanistic rodent systems with clinically representative porcine models may ultimately provide a more effective translational framework for bridging preclinical studies and human therapeutic development [11,14].

## 9. Conclusions

Experimental animal models remain indispensable tools for investigating the pathogenesis of inflammatory bowel disease (IBD) and evaluating emerging therapeutic strategies. Because IBD is a highly complex and heterogeneous disorder involving epithelial barrier dysfunction, immune dysregulation, microbiota imbalance, and chronic tissue remodeling, no single experimental system can fully reproduce all aspects of human disease. Consequently, appropriate model selection should be guided by the specific pathological and translational objectives of each study.

Rodent models continue to provide essential mechanistic insight into inflammatory signaling pathways, immune-cell regulation, epithelial injury, and host–microbiota interactions. Their accessibility, reproducibility, and well-established genetic engineering technologies make them highly valuable for molecular characterization and early-stage therapeutic screening. However, important interspecies differences in gastrointestinal anatomy, immune responses, microbiota composition, and pharmacokinetics limit direct clinical translation of rodent findings. In contrast, pig and minipig models provide substantially improved clinical value because of their close similarity to human gastrointestinal physiology, mucosal structure, immune regulation, and metabolic characteristics. These systems support procedures compatible with clinical practice, including repeated colonoscopy, serial biopsy sampling, imaging-based monitoring, pharmacokinetic analysis, and long-term therapeutic evaluation. Advances in genome-editing technologies, microbiota-associated research, and multi-omics profiling are expected to further improve the translational applicability of porcine IBD platforms. In addition, AI-assisted pathology and computational analysis may enhance objective disease assessment and biomarker discovery.

Future progress in IBD research will likely depend on integrative experimental strategies combining mechanistic rodent systems with clinically relevant large-animal platforms. Rather than replacing conventional rodent systems, pig and minipig models represent complementary translational platforms that can help bridge the gap between mechanistic discoveries and clinical application. Continued efforts to establish standardized and clinically predictive large-animal IBD models will likely improve the translational reliability of preclinical studies and support the development of more effective therapeutic strategies. The development of standardized and clinically predictive pig and minipig models capable of reproducing both UC- and CD-like pathologies will be essential for enhancing translational success in future IBD therapeutic development.

## Figures and Tables

**Table 1:** Comparative characteristics of chemically induced colitis models.

Model	Main Mechanism	Disease Feature	Strengths	Limitations	Best Use
DSS	Epithelial injury and barrier disruption	Acute or chronic UC-like colitis	Simple, reproducible, widely used	Mainly injury-driven, dose/batch variability	Drug screening, barrier function, innate immunity
TNBS/ DNBS	Hapten-mediated immune response	CD-like transmural inflammation	Strong immune activation, chronic inflammation possible	Technical variability, ethanol injury component	T-cell-mediated inflammation, fibrosis
Oxazolone	Hapten-induced skewed inflammation	UC-like Th2-superficial inflammation	Useful for Th2-type mucosal inflammation	Less commonly used, protocol-sensitive	UC-like immune response
Acetic acid	Direct chemical injury	Acute ulceration	Rapid, inexpensive	Poor chronic relevance	Acute injury and mucosal repair

Chemically induced colitis models are widely used in IBD research because of their simplicity, reproducibility, and ability to reproduce specific pathological features of intestinal inflammation. DSS-induced colitis primarily reflects epithelial barrier disruption and innate immune activation, whereas TNBS/DNBS-induced models are more suitable for studying T-cell-mediated transmural inflammation. Oxazolone-induced colitis is associated with Th2-type mucosal inflammation, while acetic acid models are primarily used for investigating acute epithelial injury and mucosal repair. Abbreviations: DSS, dextran sulfate sodium; TNBS, trinitrobenzene sulfonic acid; DNBS, dinitrobenzene sulfonic acid; UC, ulcerative colitis; CD, Crohn’s disease; Th, T helper cell.

**Table 2:** Comparative characteristics of pig and minipig models used in translational inflammatory bowel disease research.

Model	Species	Induction	Duration	Major Pathology	Endoscopy	Histology	Limitation
DSS Pig	Pig	DSS 1–1.25%	7–14 d	UC-like	Yes	Crypt loss	Variability
DSS Minipig	Göttingen	DSS	14 d	UC-like	Yes	Ulceration	Cost
DNBS Pig	Pig	DNBS intrarectal	Chronic	Transmural	Yes	fibrosis	reproducibility
TNF△ARE Pig	GM pig	Spontaneous	Chronic	CD-like	Yes	Transmural inflammation	Availability

Pig and minipig models provide enhanced clinical relevance for IBD research because of their close similarity to human gastrointestinal anatomy, immune responses, and microbiota composition. The table summarizes currently available porcine colitis models, including their induction methods, major pathological features, translational applications, and limitations. Abbreviations: DSS, dextran sulfate sodium; TNBS, trinitrobenzene sulfonic acid; DNBS, dinitrobenzene sulfonic acid; UC, ulcerative colitis; CD, Crohn’s disease; IBD, inflammatory bowel disease.

**Table 3:** Comparative features of rodent and pig/minipig colitis models in translational inflammatory bowel disease research.

Parameter	Rodent Models	Pig/Minipig Models
Cost	Low	High
Throughput	High	Low to moderate
Genetic tools	Well established	Developing
Human anatomical similarity	Limited	High
GI physiology similarity	Moderate	High
Immune similarity	Moderate	Relatively high
Endoscopy	Limited, technically difficult	Clinically feasible
Serial biopsy	Difficult	Feasible
Clinical-scale dosing	Limited	Feasible
Device evaluation	Limited	Highly suitable
Translational applicability	Moderate	High
Best use	Mechanism, screening	Late-stage preclinical validation, endoscopy, biomarker analysis, device-based studies

Rodent and porcine models provide complementary advantages for preclinical IBD research. Rodent systems are widely used for mechanistic studies and therapeutic screening because of their accessibility, reproducibility, and genetic manipulability, whereas pig and minipig models offer greater translational relevance owing to their close similarity to human gastrointestinal anatomy, physiology, and immune responses. The table summarizes the major differences between rodent and porcine colitis models with respect to experimental utility, clinical applicability, and translational value.

**Table 4:** Recommended evaluation endpoints according to experimental colitis models.

Model	Primary Pathological Feature	Recommended Endpoints	Translational Relevance
DSS	Epithelial injury and barrier dysfunction	DAI, colon length, histology, ZO-1, occludin, claudins, permeability assays	Barrier protection and mucosal healing
TNBS/DNBS	Transmural inflammation and fibrosis	Histology, fibrosis scoring, collagen deposition, TNF-α, IL-17, IFN-γ	Crohn’s disease-like pathology
IL-10 KO	Chronic immune dysregulation	Cytokine profiling, immune-cell analysis, microbiome assessment	Mechanistic immunology
Adoptive transfer	T-cell-mediated inflammation	T-cell subsets, cytokine profiling, histology	Immune-targeted therapy
Infectious/ microbiota	Host–microbiota interaction	Microbiome sequencing, barrier markers, inflammatory cytokines	Dysbiosis studies
Pig/minipig	Translational disease monitoring	Endoscopy, serial biopsy, imaging, PK/PD, blood biomarkers	Preclinical therapeutic validation

The table summarizes representative pathological features and recommended outcome measures for commonly used experimental colitis models. Selection of evaluation endpoints should be guided by the specific disease mechanisms and translational objectives of each model. Abbreviations: DAI, disease activity index; PK/PD, pharmacokinetics/pharmacodynamics.

## Data Availability

No new data were created or analyzed in this study. Data sharing is not applicable to this article.
